# Microbial allies: Enhancing plant defense via phenylpropanoid pathway and lignification

**DOI:** 10.1093/plphys/kiaf059

**Published:** 2025-03-04

**Authors:** Ritu Singh

**Affiliations:** Assistant Features Editor, Plant Physiology, American Society of Plant Biologists; Department of Plant Science, University of California, Davis, CA 95616, USA

Plants encounter a diverse range of microorganisms, including both pathogens and beneficial microbes. While pathogens trigger plant defense responses, beneficial microbes, such as certain rhizobacteria, can induce systemic resistance, priming the plant immune system to combat future threats. Similar to pathogen-induced resistance, induced systemic resistance involves a complex interplay of metabolic pathways, hormonal signaling, and immune components, characterized by the accumulation of reactive oxygen species, activation of defense-related genes, and production of pathogenesis-related proteins (Pieterse et al. 2014; De Kesel et al. 2021).

The phenylpropanoid pathway, derived from phenylalanine metabolism, is central to plant immunity, producing antimicrobial compounds like flavonoids and lignin and strengthening the plant's physical barriers (Naoumkina et al. 2010). While phenylpropanoid pathway significance is well established in pathogen-triggered defense, its role in induced resistance remains elusive. A recent study by Li et al. (2024) in *Plant Physiology* highlights the connection between phenylalanine metabolism, phenylpropanoid biosynthesis, and induced resistance. The study reveals how JR48 rhizobacteria regulate phenylpropanoid biosynthesis and lignification to bolster plant defense.

Li and coworkers (2024) observed that treatment with the rhizobacterium JR48 significantly reduced lesion diameters caused by *Phytophthora capsici*, a hemibiotrophic pathogen, on tomato, pepper, and tobacco leaves compared with untreated controls. JR48 treatment also increased hydrogen peroxide and free salicylic acid (SA) levels and enhanced lignin content in infected pepper plants, indicating that it induces resistance through a conserved mechanism.

Transcriptome analysis revealed that JR48 treatment significantly enriched defense-related pathways, particularly phenylpropanoid biosynthesis, which correlated with observed lignin accumulation during infection. Within this pathway, 16 peroxidase genes were upregulated, with 5 showing a more than 2-fold increase in expression compared with control, indicating their probable involvement in JR48-mediated immunity.

To evaluate the role of these peroxidase genes in JR48-mediated immunity, they were ectopically expressed in *Nicotiana benthamiana* and pepper leaves. Transient expression assays showed that 3 peroxidase genes—*Capana03g001836*, *Capana02g002990*, and *Capana04g001245*—significantly reduced *P. capsici* colonization and promoted lignin accumulation. Interestingly, these genes did not affect free SA levels during infection, indicating their role in resistance is SA independent. Similarly, stable transgenic *Arabidopsis* plants expressing these peroxidase genes exhibited enhanced resistance to *P. capsici* and bacterial pathogens. Additionally, loss-of-function analysis via virus-induced gene silencing in pepper plants confirmed that only *Capana03g001836* was essential for lignification and resistance to *P. capsici*, reducing lignin content and increasing lesion area upon silencing. Collectively, these findings suggest that *Capana03g001836*, *Capana02g002990*, and *Capana04g001245* positively and redundantly regulate pathogen resistance through lignification, with *Capana03g001836* playing a dominant role, likely acting downstream of SA signaling in the phenylpropanoid pathway.

To further validate the role of the phenylpropanoid pathway in JR48-induced resistance, inoculation assays were performed using *Capana03g001836*-silenced plants and plants treated with the phenylalanine ammonia-lyase inhibitor (2-aminoindane-2-phosphonic acid [AIP]). JR48 and its cell-free supernatant (CFS) treatment significantly reduced *P. capsici* infection and increased lignin content in control plants, but these effects were diminished in silenced or AIP-treated plants. These findings confirm that lignification, mediated by the phenylpropanoid pathway, is essential for JR48-induced resistance. Interestingly, JR48 and CFS elevated free SA levels in *Capana03g001836*-silenced or AIP-treated plants, indicating that phenylpropanoid-dependent lignification operates downstream of SA signaling.

Further, to identify the resistance-inducing substances produced by JR48, the authors fractionated JR48 into CFS, a filtered component (devoid of large protein molecules), and a retained component (containing protein molecules). Only CFS and filtered component treatments significantly reduced *P. capsici* infection, increased lignin content, and elevated free SA levels, suggesting that JR48 produces small molecules responsible for inducing resistance.

Nontargeted metabolomics identified phenylpyruvate, a metabolite in phenylalanine metabolism, as a key component enriched in JR48 CFS. Phenylpyruvate significantly reduced lesion sizes and enhanced lignin content during *P. capsici* infection. When combined with SA, phenylpyruvate synergistically increased lignin biosynthesis and resistance. Additionally, SA treatment alone activated peroxidase gene expression in a dose-dependent manner, an effect further amplified during pathogen infection. In contrast, phenylpyruvate alone had little impact on peroxidase gene expression, irrespective of SA treatment. These findings suggest that pathogen infection and SA treatment activate peroxidase expression, while phenylpyruvate and SA synergize to enhance lignification and resistance.

In conclusion, JR48 enhances pathogen resistance by producing phenylpyruvate, which fuels phenylalanine-dependent lignin biosynthesis and synergizes with SA signaling to activate peroxidase expression. Without JR48, plants rely on SA signaling to induce peroxidase expression and lignification-mediated resistance during infection (Fig.).

**Figure.**
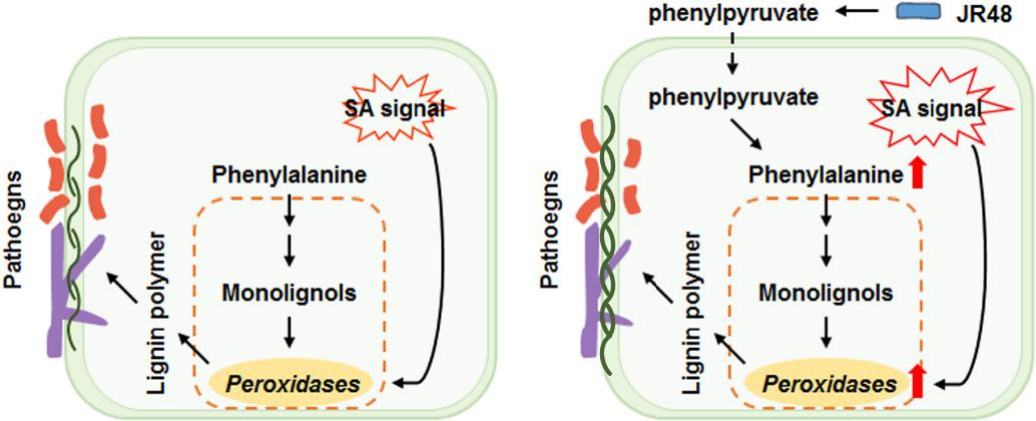
Proposed model illustrating JR48-induced plant resistance (adapted from Li et al. (2024) Figure 7D). **Left panel:** In the absence of JR48 during a pathogen attack, plants depend on SA signaling to induce peroxidase expression and lignification-mediated resistance. **Right panel:** With JR48 pretreatment, JR48-generated phenylpyruvate replenishes phenylalanine-dependent lignin biosynthesis and amplifies SA signaling, further boosting peroxidase expression and lignification. This dual mechanism significantly enhances resistance against pathogens.

## Data Availability

No data is generated in this study.

## References

[kiaf059-B1] De Kesel J, Conrath U, Flors V, Luna E, Mageroy MH, Mauch-Mani B, Pastor V, Pozo MJ, Pieterse CMJ, Ton J, et al The induced resistance lexicon: do's and don'ts. Trends Plant Sci. 2021:26(7):685–691. 10.1016/j.tplants.2021.01.001 33531282

[kiaf059-B2] Li Q, Liu Z, Jiang Z, Jia M, Hou Z, Dou D, Yu J. Phenylalanine metabolism-dependent lignification confers a rhizobacterium-induced plant resistance. Plant Physiol. 2024:197(2):kiaf016. 10.1093/plphys/kiaf016 39951289

[kiaf059-B3] Naoumkina MA, Zhao Q, Gallego-Giraldo L, Dai X, Zhao PX, Dixon RA. Genome-wide analysis of phenylpropanoid defence pathways. Mol Plant Pathol. 2010:11(6):829–846. 10.1111/j.1364-3703.2010.00648.x 21029326 PMC6640277

[kiaf059-B4] Pieterse CM, Zamioudis C, Berendsen RL, Weller DM, Van Wees SC, Bakker PA. Induced systemic resistance by beneficial microbes. Annu Rev Phytopathol. 2014:52(1):347–375. 10.1146/annurev-phyto-082712-102340 24906124

